# Notes on Army Medical Service

**Published:** 1862-08

**Authors:** Sanford B. Hunt


ART. V.—*Notes on Army Medical Service, by* SANFORD B. HUNT, M.D.

MR. EDITOR:—I comply more cheerfully than easily with your request to furnish to your journal some notes of my service on the Peninsula, extending over a period, I think, of eleven weeks. For some years past my pen has been unused to medical authorship and it may be difficult for me to resume a once easy and delightful task. I reached Fortress Monroe soon after the battle of Williamsburgh, and after serving in any capacity to which I happened to be assigned during the interval, left for home some three weeks after the retreat which brought McClellan's army to Harrison's Landing. My experience was probably more varied than that of most surgeons, but less exciting. Such professional lessons as I derived from it, I shall gladly communicate. I saw no field service whatever, but was enabled to witness its results in hospitals and transports, and feel authorized to speak with some emphasis upon it.

Gun-shot wounds, reduced to their simplest statement, are only contused and lacerated wounds, in which bones or blood-vessels may or may not be involved. The contusion is ordinarily an unimportant feature. As a musket ball may go through a pane of glass wlthout shattering it, so,—and much more so—it passes through the soft parts of the human body, leaving around it but a small contusion, practically unimportant in the treatment. The laceration, then, and the parts it involves, are the main object of the surgeon's care. From this statement it is pretty evident that each wound must be considered as an isolated fact and that all special ideas, such as poison from the powder and gangrenous effects said to be produced by the rapidity with which the missile impinges upon the parts, should be discarded. This reasoning by exclusion is not altogether unnecessary, for too many surgeons look upon a gun-shot wound as a *lusus*, to be met with extraordinary means. Really, however, a gun-shot wound is only a laceration—its importance depends upon the parts involved and the character of the missile.

In many flesh-wounds the bullet cannot be easily found. Even a minie ball will glance if it impinges at an acute angle on a firm fibrous membrane, and the result is that a ball may pursue a very tortuous course. In such instances, nothing can be gathered from the history of the case. Neither will the probe reveal the course of the missile or follow its crooked channel—consequently if no instant danger requires action, and unless the bullet can be felt from the surface, it is not worth while to go upon a very doubtful search with the scalpel. It is very true that the ragged form of the minie is much less likely to remain harmless than the round bullet, but, at least in field operations, time and careful study should be given to the case before cutting. This is especially true of all wounds of the trunk and head, which cannot be cured by amputation. In such cases, where the lungs or abdominal viscera are perforated, the patient is virtually out of the range of surgery and within that of medicine, to be treated as the indications may decide. So far I have excluded from operations all wounds in which the bullet cannot be easily found. This narrows down the duty of the scalpel to amputations of the extremities, resections of bones or the ligation of arteries.

*Amputations.*—It is universally conceded that amputations on the field are far more successful than secondary operations. The soldier does not come off the field horrified and crushed down by unexpected and sudden shock, like the victim of a railroad accident. He has his wits about him, had some notion of being hit, finds it hurt him less than he expected, and so meets the amputating knife with a nervous system capable of enduring further shock. In fact he is usually in a state of exaltation that upholds him during the ordeal. Thus it happens that very high amputations of the thigh, even, have, with proper after treatment, a fair chance of success, if performed early. But when the other course is taken, when the sufferer lies all night on the field, is borne in the morning to an ambulance, jolted over bad roads, and transferred, days afterward, to a hospital, he has become worn, jaded, incapable of endurance, irritative action has set in and the secondary amputation is fatal.

The truth of these remarks is an axiom, yet there are a vast number of cases which seem compelled to be exceptions. A bullet strikes the upper third of the femur, buries itself in the bone, perhaps without absolutely fracturing it, and the case becomes one of doubt, justifying delay. So too with wounds of the joints, especially the elbow, which somehow endures disaster better than any other joint. Such cases are those which reach the General Hospital and come under the care of its surgeons. At first, immediately after the Williamsburgh battle, the disposition in the various hospitals at Fortress Monroe was to operate. The knife was used with heroic freedom. It would be invidious to specify the surgeons who made that raid upon humanity. A bullet was looked upon as a prize worth any amount of digging for, and some ghastly and even fatal wounds were inflicted by the scalpel in a prolonged search for an unoffending pellet of lead. Amputations seemed particularly attractive, and many a man lost his thigh at the upper third, to die next day and exhibit on the post-mortem a fracture splitting the bone into the acetabulum. It is hardly necessary to say that these were cases in which no operations should have been had. The opportunity for success was lost when the surgeon on the field decided not to amputate, and in the General Hospital it only remained to extract such fragments of bone as could be readily reached, to keep the limb moderately extended, and then to give the poor fellow a chance to pass the dangers of tetanus and drag through the perils of an exhausting discharge with its irritative fever, and perhaps its purulent absorption. Truly this is a melancholy choice, but when you know that the patient will die under the knife, it is only fair to let him fight it out with Nature.

The lesser amputations, however, escaped this criticism, except when hospital gangrene supervened, as it did at the mis-named Hygiea Hospital. The result of the amputation depended on the magnitude of the tissues cut, and in arms or legs the termination was mostly favorable, excepting always hospital gangrene. It was, I believe, only in the Hygiea Hospital that this terrible scourge exhibited itself. That building was formerly a hotel inclosing a court-yard, the yard itself bisected by a long two story building, and a mouldy wooden pavement covering the ground and forming a cloaca for dampness and decay. Shaded with trees, gloomy and ill-ventilated, it was no wonder that gangrene showed itself in wards which so far as sanitary police was concerned, were well kept. The fatality from this source became so great—killing off so many of the capital operations—that Medical Director Cuyler closed the building, and had nearly evacuated the premises, when the seven days retreat, compelled its re-opening and renewed the gangrene. This hospital was therefore finally abandoned about the last of July.

The mortality from capital operations should not, however, be attributed altogether to hospital gangrene. It was quite as bad as it could be in other institutions. Some of the distinguished eastern surgeons who volunteered their services, would be troubled now to find one of their patients on this side of the Styx. One such operated largely in Mill Creek Hospital, ligating arteries, resecting bones and amputating. Of all on whom he laid his knife, not one is now alive to tell the tale of heroic surgery. I make this statement with a knowledge of its truth. About this time Brigade Surgeon John W. Hunt, a Western New Yorker, took charge at Mill Creek —Preliminary to other reforms, he carefully locked up the surgical instruments and relied on a pair of scissors and a forceps to treat several hundred wounded. He certainly killed nobody, and when the records of that hospital shall be written up, they will show a triumph of conservative surgery. Not only was the mortality largely decreased, but in hosts of cases the patients were restored with useful limbs. With Surgeon Hunt,—who, I am sorry to say, is not a relative of mine—should rank his able friend, Surgeon McCay, of the Chesapeake Hospital, a mammoth institution, unfavorably constructed and located, but nobly managed. And here let me add that a full breast of the milk of human kindness is a grand essential in an army surgeon. Hunt and McCay were kind-hearted as well as skillful and judicious.

The result of resections of bones will hardly warrant a more favorable record than I have bestowed on high amputations of the thigh. Possibly in civil practice or in city hospitals, better results might be obtained. They were not successful at Fortress Monroe, and conservative surgeons did not hesitate to condemn their frequent employment.

In the ligation of arteries, the rules of general surgery seemed strictly applicable. When great arteries are tied the parts beyond are very apt to die, yet there is far less objection to these ligations than to resections and amputations. A patient is bleeding from a deep-seated branch of the external carotid, death is imminent, and the tying of the common carotid will at least prolong, if it does not save, his life, so that the surgeon can reconcile his conscience with his knife, which, by the way, is a somewhat important, though often neglected, preliminary to any important act in military surgery.

A word should be said here about the probable proportion of operations to cases. On the field it is large of course, and I have endeavored to show that there is the place where most of the operative surgery should be done. But as cases reached the great hospitals at Fortress Monroe and Newport News, the surgeon not eager to cut, would find that the regimental surgeons had done pretty nearly all that was justifiable, and that nothing was left for him, except a large faith in nature, and a few secondary operations rendered necessary by complications occurring at a later day. They will not average one operation to a hundred cases. It remains to treat the majority *pro re nata*, to apply cerate to the kindly wounds, and water dressings to those inflamed, to watch carefully their cleanliness, to support with wines and tonics under exhausting discharges, to temper irritability with opium, and to secure for them as good a diet and as pure an air, as circumstances will permit.

All this does not accord with the picturesque idea of an army surgeon with sleeves rolled up, and up to his ankles in blood; but such pictures belong only to the battle-field, and they are far less common than the lively imagination of "Sawbones" would paint. To sum up, then, the treatment of gun-shot wounds is practically more simple than it has seemed to our unaccustomed minds. Operations for their relief are most successful upon the field, while in hospitals and under the depressing circumstances that surround them, it is the dictate of a prudent judgment to avoid, so far as possible, the use of the knife, which is in fact, unnecessary in nine cases out of ten.

In subsequent articles I shall speak of the fevers of the peninsula, of army diseases generally, of hospital construction and of the general routine of duty of the army surgeon.

---

A suit for damages in a case of fracture of the leg followed by mortification and amputation, was lately brought before the Court of Common Pleas in Franklin County, Ohio, and resulted in a verdict for the defendant, Dr. G. W. Butler. It was claimed by the defendant on the trial, that a special contract was made with plaintiff, previous to treatment, releasing the former from all responsibility as to its result; and the validity of such a contract seems to have been allowed by the Judge, and the fact of its existence left with the jury to decide.

